# An efficient swarm intelligence approach to the optimization on high-dimensional solutions with cross-dimensional constraints, with applications in supply chain management

**DOI:** 10.3389/fncom.2024.1283974

**Published:** 2024-01-18

**Authors:** Hsin-Ping Liu, Frederick Kin Hing Phoa, Yun-Heh Chen-Burger, Shau-Ping Lin

**Affiliations:** ^1^Data Science Degree Program, National Taiwan University, Taipei, Taiwan; ^2^Institute of Statistical Science, Academia Sinica, Taipei, Taiwan; ^3^Department of Computer Sciences, Heriot-Watt University, Edinburgh, United Kingdom; ^4^Institute of Biotechnology, National Taiwan University, Taipei, Taiwan

**Keywords:** supply chain management, swarm intelligence, tensor-type particle, CPU parallelization, selling scheme

## Abstract

**Introduction:**

The Swarm Intelligence Based (SIB) method has widely been applied to efficient optimization in many fields with discrete solution domains. E-commerce raises the importance of designing suitable selling strategies, including channel- and direct sales, and the mix of them, but researchers in this field seldom employ advanced metaheuristic techniques in their optimization problem due to the complexities caused by the high-dimensional problems and cross-dimensional constraints.

**Method:**

In this work, we introduce an extension of the SIB method that can simultaneously tackle these two challenges. To pursue faster computing, CPU parallelization techniques are employed for algorithm acceleration.

**Results:**

The performance of the SIB method is examined on the problems of designing selling schemes in different scales. It outperforms the Genetic Algorithm (GA) in terms of both the speed of convergence and the optimized capacity as measured using improvement multipliers.

## 1 Introduction

As technology and human knowledge have advanced, industrial and scientific investigators attempt to solve large-scale complex optimization problems that mostly fall in the category of NP-hard. The complexity comes not only from the high-dimensional solution domain that tests the computational capacity of hardware and software but also from cross-dimensional constraints. Since most traditional optimization methods are inefficient, if not infeasible, for these large-scale problems in today's real world, researchers search for new algorithms that can balance efficiency and accuracy. Metaheuristic algorithms sacrifice a part of accuracy to pursue extra efficiency and provide reasonable solutions to optimization problems using adequate resources. These algorithms typically sample a subset of the solution space that is too large to be completely enumerated. Metaheuristic algorithms can also handle multi-dimensional real-values problems without relying on the gradient of the objective functions, which enables them to search over solution spaces that are non-continuous, noisy, or changing over time.

As its primary class, nature-inspired metaheuristics can be further categorized into two main categories. Evolutionary algorithms, such as Genetic Algorithm (GA) (Goldberg, 2003), Genetic Programming (GP) (Cramer, 1985), Differential Evolution (DE) (Storn and Price, 1997), and many others, are inspired by Darwin's evolutional theory that allows only those with the fittest characteristics to survive in a competition among species members when individual variations randomly occur and are hereditary. On the other hand, swarm algorithms, such as Ant Colony Optimization (ACO) (Dorigo and Gambardella, 1997), Artificial Bee Colony (Dervis and Basturk, 2007), Particle Swarm Optimization (PSO) (Kennedy and Eberhart, 1995; Kennedy, 2010), Swarm Intelligence Based (SIB) method (Phoa et al., 2016; Phoa, 2017), and many others, mimic the collective behavior of self-organized and decentralized systems for characteristics improvements. Among all, PSO is one of the most representative swarm intelligence algorithms in engineering problems and some scientific research in the past decades. With well-defined physical meanings, it efficiently tackles high-dimensional optimization problems in continuous solution spaces, but it may not be the first choice for non-continuous solution spaces that commonly appear in mathematics, statistics, and many other fields, even via the remedy of a simple round-off (Kim et al., 2010). As a result, the SIB method (Phoa, 2017) was proposed for this manner with a wide range of applications, including the constructions of optimal experimental designs (Phoa et al., 2016), the uniform distribution of testing points (Phoa and Chang, 2016; Huang and Phoa, 2023), supercomputing scheduling (Lin and Phoa, 2019), hot spot determination (Hsu and Phoa, 2018), traveling salesman problem (Yen and Phoa, 2021), and many others.

First introduced in 1982, the concept of supply chain management (SCM) is to organize the flow of goods and services, including all processes from obtaining raw materials to delivering final products to customers. As the connection between suppliers and customers, SCM aims to minimize total costs within a supply chain and maximize a company's net profits. It increases a company's profit and gains its competitive advantage over the market. More about the basics of SCM are referred to Fredendall and Hill (2001) and Mentzer et al. (2001). Nonetheless, there is a growing gap between advanced optimization techniques and real applications in SCM. Even though advanced methods have been available in computer science and engineering for decades, many applications still use traditional optimization methods such as linear programming (Delloite, 2022). Without advanced techniques that can significantly reduce the computational cost, researchers in SCM confront difficulty in developing large-scale data analysis systems for optimizing multi-supplier selling schemes, a many-to-many network where products are delivered directly from multiple suppliers to multiple customers. Moreover, optimization in the SCM usually suffers from both the high-dimensional solution domain and cross-dimensional constraints. Without the latter constraint, the former complexity may simply be solved by decomposing the high-dimensional solution space into multiple low-dimensional ones and then optimizing each low-dimensional space once at a time. The existence of cross-dimensional constraints breaks the independency assumption among the decomposed low-dimensional domains; thus, the simple divide-and-conquer approach is no longer applicable for simplifying the problem.

This work introduces a metaheuristic optimization method via swarm intelligence that can solve high-dimensional problems and deal with cross-dimensional constraints simultaneously. Section 2 briefly reviews three nature-inspired metaheuristic optimization techniques related to our work. Section 3 introduces the implementation details of the SIB method for the optimization problem of multi-supplier selling schemes. Then, the proposed method is evaluated with several simulated supply chains on different scales and compared with the Genetic Algorithm (GA) in Section 4. Finally, some conclusions are in the last section.

## 2 Nature-inspired metaheuristics optimization methods

### 2.1 Genetic algorithm

The Genetic Algorithm (GA), proposed in Holland (1975), is one of the oldest and the most popular nature-inspired metaheuristic algorithms. Based on the mechanics of the natural selection procedure, it follows the concept of “the survival of the fittest,” where the stronger individuals tend to survive while the weak ones approach extinction. Like many other metaheuristics, GA starts with a population consisting of a group of particles. A particle, called a “chromosome” or a “genotype,” represents a possible solution to the target problem, and its parameters are called “genes.” Each iteration, known as a generation, comprises crossover, mutation, and survivor selection to simulate the hereditary phenomenon. Moreover, to implement the survival of the fittest concept, there is a parent selection at the beginning of each iteration.

After randomly selecting an initial population from the solution space, the objective function evaluates the particles and ranks them by their performances. The particles with higher ranks are considered in the candidate pool for parent selection. With two or more particles selected from the pool, a crossover randomly exchanges their genes, which results in one or two children particles. In addition, a mutation occurs randomly on the particles in each iteration to mimic random genetic perturbations. At the end of an iteration, some lower-rank particles are eliminated to maintain the size of the population (survivor selection). This iterative process continues until the fulfillment of the pre-defined stopping criterion, and the particle with the top rank is the optimized output of GA.

### 2.2 Particle swarm optimization algorithm

PSO (Kennedy and Eberhart, 1995; Kennedy, 2010) is prevalent in many industrial and scientific optimization fields due to its easy implementation and efficiency in terms of memory and speed. It is designed to mimic the social behavior of a flock of birds, which contains a leader and several members. While the leader's movement affects all the members (group effect), each member has their individual thoughts about their own movement (personal effect). In a PSO algorithm, an initial swarm, consisting of several particles, corresponds to the initial state of a flock, and the position of a particle represents a possible solution to the target optimization problem. In addition, Local Best (LB) particles and the Global Best (GB) particle are determined based on a pre-defined objective function. Each particle has its own LB, which is the best position it has encountered so far, while the GB is the best solution the whole swarm has encountered.

A velocity is assigned to a particle for implementing the group and personal effects. The position of a particle is influenced by its LB and the GB through its velocity in each iteration. The updating formula of a velocity and a position can be expressed as the following equations:


$$\begin{array}{l} v_{i}^{t+1}\leftarrow av_{i}^{t}+b\left(x_{i}^{lb,t}-x_{i}^{t}\right)+c\left(x_{i}^{gb,t}-x_{i}^{t}\right)\\ x_{i}^{t+1}=x_{i}^{t}+v_{i}^{t}, \end{array}$$

where *t* denotes the number of iterations, *i* indicates the number of dimensions, *v* is the velocity of the particle, *x* is the particle's position, *x*^*lb*^ is the LB position of the particle, *x*^*gb*^ is the GB position of the swarm, and *a, b, c* are scalars. A velocity consists of three parts corresponding to the inertia, the personal effect, and the group effect. The three scalars indicate the weight given to each part. The update process continues until a user-defined termination criterion is fulfilled, and the final GB position is the optimized output.

### 2.3 Swarm intelligence based algorithm

The SIB method (Phoa et al., 2016; Phoa, 2017) can be considered a hybrid algorithm that takes advantage of both the GA and the PSO. Specifically, it preserves the general framework of the PSO that includes the initial group of particles, the local best and group best, and the communication among particles after the updates (called MOVE operation in SIB). In order to adapt to the discrete nature of the solution domain, SIB gives up the velocity and position updates in the PSO and embraces the update procedures like crossover (called MIX operation in SIB) and mutation (called random jump in SIB).

In specific, after initializing a swarm, SIB enters an iteration loop consisting of MIX, MOVE, and a stopping criterion. In the MIX operation, every particle is mixed with its own LB and the GB, which returns two new particles, called *mixwLB* and *mixwGB*, respectively. To “mix” a particle with the best particle, a given proportion of entries is modified according to the corresponding values in the best particle. The implementation of this operation is flexible and can be designed according to the target optimization problem. It is a rule-of-thumb to allow a smaller proportion of entries to be modified by the global best particle than by the local best particle to avoid premature convergence without sufficient domain explorations. Once the new particles are generated, the MOVE operation decides which one of the three particles, the original particle, *mixwLB*, and *mixwGB*, is selected as the update. It is straightforward to update the particle if either of the mixed particles has the best objective function value. If the MIX operation fails to improve the original particle, a random jump, in which a given proportion of entries are altered randomly to create a new particle, is operated to avoid trapping in the local optimum.

Some stopping criteria for the algorithm should be defined beforehand. Most of the time, the target problem comes with adequate stopping criteria. If there is no specific one, a maximum number of iterations and convergence toward a pre-defined threshold range of GB are common choices of stopping criteria.

## 3 Method and implementation

As a popular selling strategy in E-commerce nowadays, multi-supplier selling is a process of selling products from many suppliers to many customers. This strategy is a kind of direct sales with no dealers or intermediaries in the selling scheme, and products are sold and delivered to the customers directly from the suppliers. A direct sale market structure not only increases suppliers' profits and decreases the prices of products for customers but also simplifies the complexity of the target optimization problem. However, even with a direct sale, the optimization task on multi-supplier selling schemes is still complicated due to high-dimensional solution space and cross-dimensional constraints. Thus, the SIB method is chosen to solve this optimization problem.

**Figure 1:**
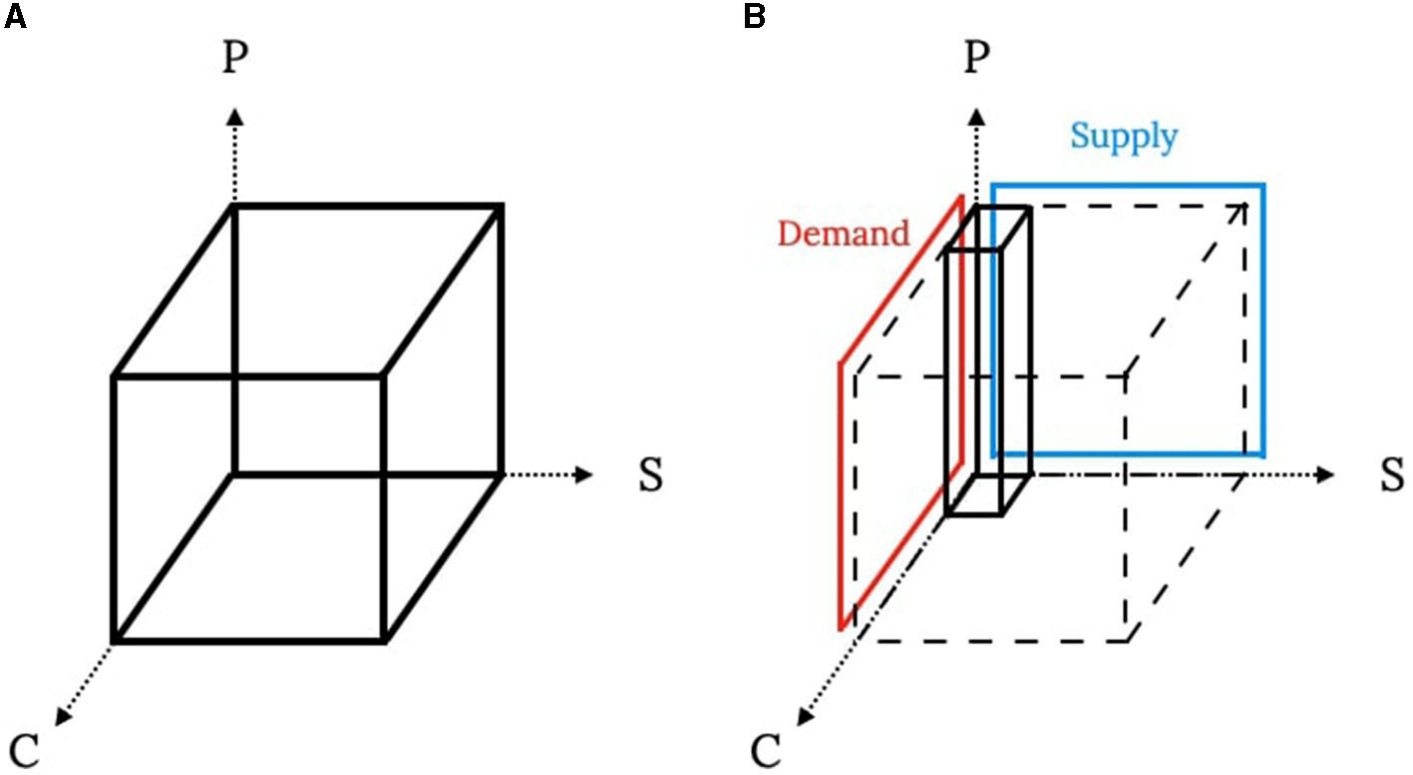
Visualization of particle definition and constraints. **(A)** Particle definition. **(B)** A column and constraints.

Denote a selling scheme with *N* customers, *K* product types, and *M* suppliers, and a tensor *X* with dimensions *N*×*K*×*M* to represent this selling scheme. Figure 1A illustrates the definition of a particle while the *C*-, *S*-, and *P*-axes correspond to customers, suppliers, and product types. Each entry *x*_*nkm*_ indicates the number of the *k*th product sold to the *n*th customer by the *m*th supplier, and each column (Figure 1B) with *K* entries indicates the selling scheme between a customer and a supplier.

The following assumptions are made for this work. First, the quantities of supply and demand are known in advance, and the supply is less than the demand to create a more challenging situation where prescriptive analytics is needed. Second, no further complications on resale, buy-back, or others exist. Third, customers are willing to purchase an identical product from multiple suppliers. Finally, each customer pays a constant price for each product, called the willingness to pay in economics. In real markets, a shortage in supply, which is the first assumption, is likely to make customers pay the maximum prices they are willing to pay.

In a SCM task, the objective function is generally the profit that a selling scheme can make, equal to the difference between sales and costs. The sales component is the products' prices multiplied by their quantities. Among all potential costs, this work only considers delivery and purchase costs. To calculate the delivery cost, participants of a supply chain are categorized into two geographical locations: North and South. The transportation cost per product between a specific combination of locations is a constant. The purchase cost per product from a specific supplier is also a constant. Mathematically, the objective function can be written as


$$\begin{array}{r} Profit=Sale-Cost\\ Sale=\sum \left(Quantity\times Price\right)\\ Cost=\sum \left(Delivery+Purchase\right) \end{array}$$

A supply constraint is that a supplier must have a maximum production capacity, and a demand constraint is that a customer must have the desired quantity for each product. Both constraints are cross-dimensional in nature.

Table 1 is the pseudo-code of the proposed SIB algorithm for the SCM optimization. In the initialization part, a set of valid selling schemes (particles) are randomly generated and evaluated by the objective function. Then, the initial LBs are defined as the initial positions, and the GB is the best among all LBs. In the iteration part, the MIX operation generates new selling schemes by mixing current selling schemes with the best ones, and the MOVE operation picks the best among three candidate schemes for update if the newly generated schemes provide better objective function values, or a perturbed scheme via random jump otherwise. The iteration continues until the pre-defined stopping criteria are fulfilled, which can be the maximum number of iterations, achieving a pre-defined objective function value, or converging with pre-defined rules. The final GB is considered the optimal multi-supplier selling scheme suggested by the proposed SIB algorithm. The following subsections provide a more detailed description of every step of the SIB method.

**Table 1:** The SIB algorithm.

1:	Initialize a swarm of particles.
2:	Evaluate the objective function values of each particle.
3:	Determine the Local Best (LB) and the Global Best (GB) for each particle.
4:	while STOPPING CRITERIA NOT FULFILLED
5:	Do MIX operation.
6:	Do MOVE/Random Jump operation.
7:	Update the LB and the GB particles.
8:	Check the conditions of convergence.

### 3.1 Initialization

Figure 1B shows the demand and supply constraints in this problem. To implement these cross-dimensional constraints in the algorithm, most operations are designed in a column-by-column fashion with continuous tracking of remaining demand and supply quantities. Specifically, an *N*×*K* matrix records the remaining demand, and an *M*×*K* matrix records the remaining supply. In the initialization part, each column is generated separately and combined into a complete particle. The entries in a particle are random integers chosen from 0 to the minimum between the remaining supply and demand of the target entries. Notice that the remaining quantity matrices must be updated after generating each slice of a particle. The entry should be 0 when the remaining supply or demand is 0. To increase the variations among initial particles, the generating order is shuffled in both the column and slice levels. At the end of the initialization, the best particles are set according to the particles' objective function values.

### 3.2 Iteration

#### 3.2.1 MIX operation

Each particle is mixed with its own LB and the GB for each iteration, which returns two particles denoted as *mixwLB* and *mixwGB*, respectively. Similar to the initialization, the MIX operation is in a column-by-column fashion with two remaining quantity matrices. At the beginning of a MIX operation, the remaining quantity matrices are calculated based on the original particle. A pair of columns, one from the original particle and another from the best particle (either the LB or the GB), is dealt with at a time. For each pair, entries are examined if their values in the original particle are smaller than those in the best particle and if the corresponding remaining demand and supply are both positive. In other words, if an entry has no remaining quantity in either demand or supply, it will not be modified in this operation. Then, a given proportion (*q*_*LB*_ or *q*_*GB*_) of those identified entries are randomly chosen, and the values in the original particle are replaced with the corresponding values in the best particle. In the experiments (Section 4), *q*_*LB*_ is 0.6 and *q*_*GB*_ is 0.4. While only entries with larger values in the best particle can be selected, the objective function value will only increase or remain the same in this process, and this condition is added to achieve convergence faster.

#### 3.2.2 MOVE and random jump operation

The implementation of the MOVE operation is identical to the standard SIB algorithm. The two particles, *mixwLB* and *mixwGB*, given by the MIX operation, are evaluated by the objective function, and their performances are compared to that of the original particle. If either of the mixed particles outperforms the original particle, the particle's new position is the best among the three particles. If the original particle has the best objective function value, a Random Jump operation must be executed. The Random Jump operation also uses remaining quantities matrices and column-by-column fashion. However, in the Random Jump operation, the product quantities assigned by the column should be released first, i.e., the values are added back to the remaining demand and supply. This action enhances the capability of Random Jump to bring a particle out of a local attractive trap. After calculating the remaining quantity matrices, half of the entries with non-zero demand in a column are chosen and assigned with a random integer between 0 and the minimum within the remaining demand and supply.

### 3.3 Acceleration by CPU parallelization

An advantage of the SIB method is its parallelizability, which is important for the proposed SIB method due to the time-consuming computation property among tensors. The CPU parallelization techniques are implemented with the Python package Multiprocessing for algorithm acceleration. While the data, including positions and profit values, of pairs of particles and their LB is stored in different CPUs, the data of the GB is stored in the shared memory for easy access from every individual CPU. When the MIX and MOVE operations are performed, the particles are assigned to different CPUs, and the outcomes of MOVE operations are compared with the GB individually. The data of GB will only be modified when the outcome particles perform better than the GB. However, false results may occur when multiple CPUs try to modify the data in the shared memory simultaneously. To avoid this common issue in parallel computing, a Lock is used to protect the data in the shared memory. Moreover, to keep the process synchronous, Barriers hold the complete sub-process until all the others are completed.

## 4 Experiment and result

The proposed SIB method has been applied to the small single-supplier and large multi-supplier supply chains (Phoa et al., 2021). This section evaluates the SIB method with supply chains in different scales and compares the performance with the GA algorithm. Moreover, the effect of the parallelization technique will be illustrated by showing the execution time.

To test the algorithm's general performance, supply chains' configurations in 11 different scales, including one-to-many scale, are used to set up several experiments; the detailed settings of the scales are listed in Table 2. For each scale, ten configurations are manually generated, containing different demand and supply constraints and different geographical locations for suppliers and customers. Specifically, there are some simulated supply chains in the egg market. In addition to the category and location details of suppliers and customers, the data consists of the supply amount of each supplier, the demand amount of each customer, the type of product that each supplier supplies, the cost of purchasing products from suppliers, the transport cost per mile for each egg, and product prices that are different among customers.

**Table 2:** The setting of scales.

**Scale no**.	**1**	**2**	**3**	**4**	**5**	**6**	**7**	**8**	**9**	**10**	**11**
Suppliers	1	10	20	30	40	50	60	70	80	90	100
Customers	10	10	20	30	40	50	60	70	80	90	100
Product types	3	3	3	6	6	6	8	8	8	10	10

A specific metric is used to evaluate the optimization results and to compare the results among different configurations and scales. Singh et al. (2022) defined an improvement multiplier that can measure the progress of an algorithm from the initial random values to the end of the iterations. The following equation can calculate the improvement multiplier:


$$\begin{array}{l} I=1+\frac{\left(obj_{f}-obj_{0}\right)}{obj_{0}} \end{array}$$

where *obj*_*f*_ is the objective function value of the optimized outcome, and *obj*_0_ is the objective value of the best individual among all the initial particles.

**Figure 2:**
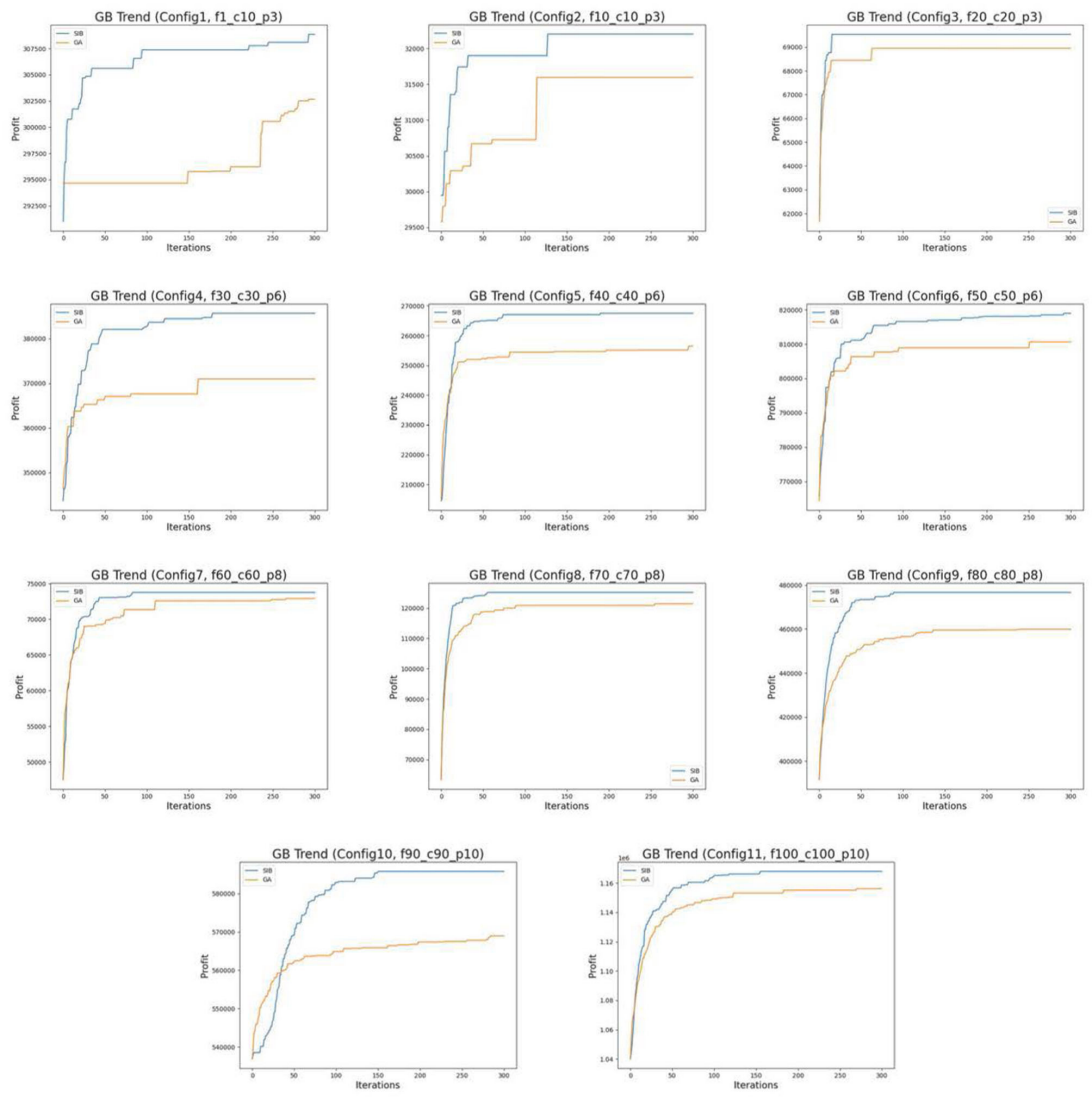
Progress trend of GB's profit.

In the first experiment, ten initial particle sets are generated based on each configuration and used as initial swarms in the GA and SIB algorithms for a fair comparison. Each algorithm is run for 300 iterations without stopping early, and the performance of the two algorithms is compared at different iterations. Table 3 shows the optimized results of two algorithms on each scale after 100, 200, and 300 iterations. According to the results, the SIB method outperforms the GA in all scales. The mean of the improvement given by the SIB is a multiple of that given by the GA, like over 1.7x, 2.5x, and 1.8x respectively in Configs. 1, 2, and 4. Furthermore, the SIB method improves faster than the GA in the early stage and converges. The means of the improvement multipliers given by the SIB in all the scales have already outperformed the GA's results after 100 steps and become stable in the rest of the steps. In contrast, the GA results start at a lower level and keep increasing until the 200th step or even the 300th step. The profit progress trend of one experiment on each scale is visualized in Figure 2, which shows our observations in an easy-understanding way.

**Table 3:** Statistics of improvement multiplier after 100, 200, and 300 steps.

**Scale no**.		**1**	**2**	**3**	**4**	**5**	**6**	**7**	**8**	**9**	**10**	**11**
**After 100 steps**												
Mean	SIB	**0.217**	**0.030**	**0.071**	**0.045**	**0.142**	**0.157**	**0.342**	**0.274**	**0.398**	**0.315**	**0.164**
	GA	0.119	0.010	0.056	0.025	0.107	0.124	0.332	0.253	0.334	0.241	0.116
Std.^a^	SIB	0.054	0.008	0.007	0.003	0.004	0.010	0.013	0.010	0.008	0.016	0.002
	GA	0.066	0.008	0.007	0.002	0.005	0.010	0.011	0.009	0.006	0.014	0.003
**After 200 steps**												
Mean	SIB	**0.222**	**0.031**	**0.071**	**0.047**	**0.144**	**0.159**	**0.349**	**0.279**	**0.408**	**0.324**	**0.167**
	GA	0.125	0.011	0.057	0.026	0.110	0.130	0.341	0.259	0.344	0.249	0.123
Std.^a^	SIB	0.055	0.008	0.007	0.003	0.004	0.010	0.012	0.011	0.007	0.016	0.002
	GA	0.070	0.008	0.007	0.002	0.004	0.010	0.012	0.008	0.007	0.013	0.006
**After 300 steps**												
Mean	SIB	**0.224**	**0.031**	**0.071**	**0.047**	**0.145**	**0.160**	**0.350**	**0.280**	**0.409**	**0.324**	**0.167**
	GA	0.130	0.012	0.058	0.026	0.111	0.133	0.345	0.261	0.347	0.252	0.127
Std.^a^	SIB	0.055	0.008	0.007	0.003	0.004	0.010	0.012	0.010	0.007	0.016	0.002
	GA	0.058	0.007	0.007	0.002	0.004	0.010	0.012	0.009	0.007	0.013	0.005

^a^The standard deviations of centralized improvement multiplier. Notice that, corresponding to ten initial particle sets, we have ten improvement multipliers for each run with each configuration. First, these ten improvement multipliers are centralized on the same run by subtracting their mean. Then a total of 100 improvement multipliers are collected for the same configuration. Finally, the standard deviation among those 100 values is reported. Bold values indicate the best values in comparison.

The second experiment tests the effect of parallel computing for large data scales. Two versions of the SIB method are implemented, one with the parallelization techniques and one without. Three configurations, No. 1, No. 4, and No. 8, are chosen to test the effect's difference in data scales. The experiments are individually run on a server with 104 cores. Table 4 summarizes the computation time in seconds. With a significantly smaller mean, the parallelization helps reduce the computing time in all three configurations. While the computing time only reduces to one-third of the time without parallel on configuration No. 1, the parallelization reduces the computing time to almost one-fifth of the time without parallel on configuration No. 8. This may result from the complexity of the computation in terms of the particle size. Since parallelization costs additional time to copy data from one CPU to another CPU, it is worthier to use this technique in experiments with higher computation complexity.

**Table 4:** Computing time (seconds).

	**No CPU parallelization**					**CPU parallelization**				
**Config**.	**Mean**	**Std**.	**Min**	**Median**	**Max**	**Mean**	**Std**.	**Min**	**Median**	**Max**
1	7.316	**0.015**	7.296	7.317	7.348	**2.353**	0.121	2.176	2.336	2.558
4	541.46	41.17	471.34	539.47	596.72	**38.30**	**0.54**	37.27	38.36	38.96
8	2891.6	109.0	2742.2	2895.2	3071.9	**639.2**	**2.8**	634.1	639.2	644.2

Bold values indicate the best values in comparison.

## 5 Conclusion

This paper proposes a SIB method to handle the multi-supplier-multi-customer supply chain management problem. A modified MIX operation is designed to handle the high-dimensional solutions; the remaining quantity matrices that store additional information help to handle cross-dimensional constraints. Moreover, parallelization techniques accelerate the program to obtain the desired results within a reasonable time. The experiments show that the SIB method, compared to the GA method, offers better-optimized solutions in a shorter time. One must consider more practical factors if this method is applied. For example, prices and costs in the real world vary based on the quantities of demand and supply, and they require a predictive marketing model before optimization. Moreover, the proposed method is not limited only to the SCM optimization problems, but also to similar optimization problems with high-dimensional domains and cross-dimensional constraints.

## Data availability statement

The original contributions presented in the study are included in the article, further inquiries can be directed to the corresponding author.

## Author contributions

H-PL: Formal analysis, Methodology, Software, Visualization, Writing – original draft. FP: Conceptualization, Data curation, Formal analysis, Funding acquisition, Investigation, Methodology, Project administration, Resources, Software, Supervision, Validation, Visualization, Writing – original draft, Writing – review & editing. Y-HC-B: Conceptualization, Data curation, Investigation, Project administration, Resources, Writing – review & editing. S-PL: Conceptualization, Investigation, Project administration, Resources, Writing – review & editing.
